# The dominant findings of a recessive man: from Mendel’s kid pea to kidney

**DOI:** 10.1007/s00467-023-06238-9

**Published:** 2023-12-05

**Authors:** Kálmán Tory

**Affiliations:** 1 MTA-SE Lendület Nephrogenetic Laboratory, Hungarian Academy of Sciences, Budapest, Hungary; 2 Pediatric Center, MTA Center of Excellence, Semmelweis University, Budapest, Hungary

**Keywords:** Mendel, Mendelian, Monogenic, Penetrance, Oligogenic, Inheritance, Genetics

## Abstract

The research of Mendel, born two centuries ago, still has many direct implications for our everyday clinical work. He introduced the terms “dominant” and “recessive” characters and determined their 3:1 ratio in the offspring of heterozygous “hybrid” plants. This distribution allowed calculation of the number of the phenotype-determining “elements,” i.e., the alleles, and has been used ever since to prove the monogenic origin of a disorder. The Mendelian inheritance of monogenic kidney disorders is still of great help in distinguishing them from those with multifactorial origin in clinical practice. Inheritance of most monogenic kidney disorders fits to Mendel’s observations: the equal contribution of the two parents and the complete penetrance or the direct correlation between the frequency of the recessive character and the degree of inbreeding. Nevertheless, beyond the truth of these basic concepts, several observations have expanded their genetic characteristics. The extreme genetic heterogeneity, the pleiotropy of the causal genes and the role of modifiers in ciliopathies, the digenic inheritance and parental imprinting in some tubulopathies, and the incomplete penetrance and eventual interallelic interactions in podocytopathies, reflect this expansion. For all these reasons, the transmission pattern in a natural setting may depend not only on the “character” but also on the causal gene and the variant. Mendel’s passion for research combined with his modest personality and meticulous approach can still serve as an example in the work required to understand the non-Mendelian universe of genetics.

Genetic research has revolutionized our understanding of the etiology, pathophysiology, and inheritance of kidney disorders during the last decades. The path from domesticating animals to the recent identification of hundreds of causal genes in monogenic kidney disorders was nevertheless long. One of the most important steps in this evolution was to understand that binomial traits are determined by two alleles of a gene, thanks to the work of Johann Gregor Mendel. Over 200 years after his birth of July 20, 1822, in Heinzendorf, Silesia, it is a good time to contemplate the influence of his observations on pediatric nephrology.

Humans have been domesticating animals for 12 millennia by selecting advantageous traits to be passed on to successive generations [1]. Thus, heredity of various traits has been known and consciously utilized for a long time by mankind. How transmission of traits from one generation to another occurs was nevertheless a matter of debate even in the nineteenth century.

The problem was formulated by Cyrill Franz Napp, the abbot of the Augustinian monastery in Brno (known as Brünn in German) between 1824 and 1867 as follows: “The question for discussion should not be the theory and process of breeding, but what is inherited and how?” [2]. Napp was also the one who welcomed Gregor Mendel into the Augustinian community as a young novice, little knowing that many years later Mendel would provide the answer to his fundamental question.

At this time, it was already recognized that all living organisms are made up of cell(s) and that during fertilization a specific cellular organelle (named nucleus by Robert Brown in 1833 [3]) is indispensable. However, the real significance and role of Brown’s nuclei were not yet clear.

The questions of this period are well reflected in the speculation of the Czech physiologist Jan Evangelista Purkyně (Purkinje, 1787–1868) about fertilization in 1834 when his wife was pregnant with their second son. He suggested that “parental characteristics are reduced in the’germs’ to’pure quality’ by a process he defined as’involution’… The meeting of the parental germs leads to’evolution’ in which the embryo develops to a form revealing the traits of both parents” [2, 4]. Purkinje was the first head of the first Physiology Department in the world in Breslau (now Wrocław, Poland). He visited the Augustinian convent on several occasions between 1835 and 1850, and may have thus influenced the work of the young Mendel [2].

## The prerequisites of Mendel’s results: the thoroughly planned research design

Crossbreeding experiments had been carried out by many predecessors of Mendel. The British Thomas Andrew Knight studied a variety of plants using artificial pollination, including the garden pea, and made similar observations to Mendel [5]. What made Mendel’s work unique is the meticulous elaboration of his research method, which allowed him to perform calculations and gave him reproducible results. As Mendel wrote in 1865: “The value and utility of any experiment are determined by the fitness of the material to the purpose for which it is used” [6]. He selected the pea (*Pisum sativum*) because it possesses easily recognizable physical characteristics; its flowers are predominantly self-pollinating, i.e., well protected from foreign pollens; it has a relatively short life cycle and is easily cultured. Furthermore, its artificial fertilization, though painstakingly elaborate, was nearly always a successful process.

Once the pea was selected, Mendel devoted 2 years selecting the traits to study. Brilliantly, out of the 34 initially studied characteristics, he excluded those which had a “‘more or less’ nature” and focused only those that “stand out clearly and definitely” and were uniform in the hybrids [6]. Selecting such binomial traits was essential for Mendel to achieve his results later.

To study the effects of inheritance as purely as possible, Mendel had to successfully overcome a number of biasing factors: natural selection, foreign pollination, and the major enemy, the pea weevil (*Bruchus pisi*). He even used controls: “For each experiment a number of pot plants were placed during the blooming period in a greenhouse, to serve as control plants for the main experiment in the open as regards possible disturbance by insects.” To counterbalance the effect of natural selection, he lifted the undersized plants and transferred them to a special bed. “This precaution was necessary, as otherwise they would have perished through being overgrown by their tall relatives” [6]. Finally, he covered the flowers with paper bags to prevent unwanted fertilization.

## Introduction of the terms ‘dominant’ and ‘recessive’

For all seven selected traits, Mendel crossed peas that differed in a single trait and found that the offspring of the two pure lines — the “hybrids” — were uniform. Moreover, not only were they uniform but they all exhibited the same trait as one of the parental plants. “In the case of each of the seven crosses the hybrid-character resembles that of one of the parental forms so closely that the other escapes observation completely…” [6]. Mendel introduced the terms “dominant” and “recessive” to describe the “power relations” of the two forms: “Henceforth in this paper those characters which are transmitted entire, or almost unchanged in the hybridization, and therefore in themselves constitute the characters of the hybrid, are termed the *dominant*, and those which become latent in the process *recessive*. The expression *recessive* has been chosen because the characters thereby designated withdraw or entirely disappear in the hybrids, but nevertheless reappear unchanged in their progeny.” The terms “dominant” and “recessive” were thus initially introduced to typify the phenotypes. In medical terms, both the disease phenotype and the disease-causing allele can be labelled dominant (over the wild type), but only the phenotype may be transmitted in a dominant fashion. Each allele is transmitted with a 50% probability to the offspring, and the disease (and not the allele) is transmitted in function of the allele’s dominant or recessive nature.

## The equal contribution of the two parents

In order to compare the parental roles in phenotype determination, Mendel performed reciprocal crossings. He found that it was irrelevant whether the dominant character belonged to the seed or to the pollen plant, and thus concluded that each sex contributes equally to the characteristics of the offspring [6]. In perfect accordance with Mendel’s observations, there is no difference between paternal and maternal origin in determining the risk of most of the kidney diseases, since the two alleles of the causal autosomal genes are in general equally expressed. Some autosomal genes are nevertheless expressed predominantly or even exclusively from one allele, which can be either randomly assigned [7] or parental-specific, as recognized in Prader-Willi and Beckwith-Wiedemann syndromes [8, 9]. Parental imprinting can lead to unequal parental effect, like the kidney abnormalities linked to the maternal loss of the 11p15.5 region in Beckwith-Wiedemann syndrome [10], or the resistance to parathyroid hormone in the proximal tubules, mainly due to the loss of the maternal *GNAS* expression or function in pseudohypoparathyroidism type I [11–13].

## From the 3:1 ratio to diploidy

Mendel was the first who concentrated on the ratio of the different forms that appeared among the offspring of the hybrids after self-pollination. His method was meticulous and precise enough to properly determine the ratio of the recessive form that reappeared in the second generation. This is how he documented the famous 3:1 ratio of plants exhibiting dominant and recessive trait, which was the same in the case of all seven traits [6]. The dominant form never reappeared in the progeny of plants with the recessive form, even after six generations of self-pollination, indicating that they do not contain any dominant “‘elements,” and thus, the recessive form only appears if the “embryo is formed from two similar cells,” i.e., if only recessive ‘elements’ are present in the parental generation [6]. It could be thus posited that if the characters (1) are transmitted unchanged to the hybrids and to their progeny, (2) the two parents contribute equally to the offspring, (3) the recessive phenotype cannot appear unless only recessive alleles are present, and (4) the recessive phenotype is present in ¼ of the offspring of the hybrid generation, then the number of the phenotype-determining “elements” (= alleles) must be two, since (½)^*n*^ = ¼, where ½ is the chance of inheriting the recessive allele in the offspring of the hybrids (which contain the two alleles in equal quantity), ¼ is the proportion of the recessive phenotype among the offspring of the hybrids, and “n” is the number of the alleles. Accordingly, Mendel used two letters as symbols of alleles, to demonstrate the crossing [6]. The Mendelian ratio thus became a synonym of the 3:1 distribution, and indicates that the character is determined by a single gene of a diploid organism. Whenever a new animal model of an autosomal recessive disease is established, the first step is to check the Mendelian ratio of the disease phenotype among the offspring of the heterozygous “hybrids” to confirm monogenic transmission, complete penetrance, and to exclude in utero lethality.

## The changing face of dominance

Mendel crossed “pure” lines, each likely to be homozygous for the associated allele. One of the two alleles was dominant over the other, as shown by the phenotype of the hybrids. In the natural setting of Mendelian disorders, each gene has thousands of pathogenic and benign alleles. Despite the heterogeneity of alleles, they are similar in their “behavior”: pathogenic alleles of a gene responsible for an autosomal dominant disorder tend to be dominant over the wild-type allele. As always, there are exceptions. A dominant disorder may turn into recessive when hypomorphic variants in a gene of an autosomal dominant (AD) disorder do not cause symptoms on their own, but only when present on both alleles. Such an example is the *PKD1* R2220W- or R3277C-associated “ADPKD” [14, 15]. These variants are recessive compared with the wild-type allele, and the severe PKD caused by them follows an autosomal recessive (AR) transmission pattern.

It is less likely that a typical AR disorder becomes dominant, as most AR disorders develop as a result of a loss-of-function that is rarely severe enough when only one of the two alleles is mutant. This has nevertheless been observed in some nephropathies, like the *IFT140*, *NEK8*, or *NPHS2*-associated ones. While their biallelic loss-of-function variants cause Mainzer-Saldino syndrome [16], renal-hepatic-pancreatic dysplasia [17–19], or steroid-resistant nephrotic syndrome [20], respectively, monoallelic variants of *IFT140* and *NEK8* were found causal in rare forms of ADPKD [21–23] and those of *NPHS2* in focal segmental glomerulosclerosis (FSGS) [24]. The monoallelic variants were specific in *NEK8* (i.e., p.R45W) [21, 22] and in *NPHS2* (p.L330Vfs*15 with a premature stop in the last exon) [24], suggesting a dominant negative effect [21], but the *IFT140* variants in mild ADPKD are intriguingly loss-of-function variants [23]. Such transformations of dominant or recessive phenotypes would have reversed the two forms of a trait in Mendel’s experiment, but would not have changed the 3:1 ratio of the dominant and recessive forms in the second generation.

The dominant/recessive nature of a variant could thus far be determined on the basis of their interaction with the wild-type allele. However, a variant that is dominant over one allele can be recessive compared with another one. An example for such interactions between alleles causing human disorders is the *NPHS2* R229Q that is benign in the homozygous state [25], recessive compared with the A284V and dominant over the R138Q pathogenic variant. As a result, the [R229Q];[A284V] combination is pathogenic and the [R138Q];[R229Q] is benign [26, 27]. Interallelic interactions which render benign variants pathogenic change not only the priority of the alleles but also the ratio of the recessive and the dominant phenotype among the offspring of the heterozygous individuals. Accordingly, healthy individuals who are compound heterozygous for a benign and a pathogenic allele: [R138Q];[R229Q] and [wt];[A284V] may have offspring that show a 1:1 proportion of the healthy and diseased phenotype instead of the Mendelian 3:1.

## Monogenic vs. polygenic

The seven phenotypic characters selected initially by Mendel were all monogenic [6]. His first observations thus corresponded to the monogenic inheritance, and “Mendelian” has become the synonym of monogenic in genetics. Later on, however, he also found characters, e.g., the flower colors of the runner bean (*Phaseolus multiflorus*) which did not conform to the 3:1 ratio:”…from the union of a white and a purple–red coloring a whole series of colors results, from purple to pale violet and white, the circumstance is a striking one that among 31 flowering plants only one received the recessive character of the white color.” Mendel nevertheless recognized that the variable characters can also be explained by the combined effect of multiple “characters”: “Even these enigmatic results, however, might probably be explained by the law governing *Pisum* if we might assume that the color of the flowers is a combination of two or more entirely independent colors, which individually act like any other constant character in the plant” [6].

Accordingly, whenever the transmission rate of a human disorder is not Mendelian, i.e., siblings with the same genotype at the primary locus present with discordant phenotypes, such as in *PLCE1*-associated nephrotic syndrome [28–30], an oligogenic inheritance can be assumed. Positing such inheritance is straightforward, but the identification of the additional loci can be challenging.

Digenic inheritance in kidney diseases has been rarely found. This would mean that the defect of a single gene is not pathogenic, but that of two is pathogenic with a complete penetrance. The hearing impairment associated with classic Bartter syndrome follows this inheritance pattern: patients with biallelic *CLCNKB* mutations develop hearing impairment if they also harbor biallelic mutations in the neighboring *CLCNKA* gene [31]. Hearing impairment develops only when the function of *both* chloride channels, encoded by the two genes, is lost. Mutations of both *CLCNKB* and *CLCNKA* also aggravate the severity of the tubular reabsorption defect, but this latter is penetrant already if any of the two genes is altered.

A similar inheritance was suggested in Bardet-Biedl syndrome (BBS) [32]. In a few families, biallelic mutations of a BBS gene were found to be penetrant only in siblings with a heterozygous mutation in a second BBS gene, leading to the proposition of “triallelic inheritance” [32]. Since several subsequent studies failed to confirm the widespread existence of this phenomenon, BBS is considered in the clinical practice to be a Mendelian, autosomal recessive disease [33–37].

A digenic inheritance was also described in Alport syndrome, even though the mutation in the second locus alters only the severity and the transmission risk of the disorder, but — apparently — not the penetrance [38, 39].

An oligogenic inheritance has been suggested in several extra-renal involvements, like the *NPHP1*-associated retinopathy and Joubert syndrome [40, 41], and even in less frequent subtypes of nephronophthisis itself [42]. While the extra-renal phenotype is clearly incompletely penetrant in siblings [40], nephronophthisis itself is considered to be autosomal recessive in clinical practice.

Overall, a causal oligogenic inheritance was more often suspected than actually demonstrated. Several misconceptions lay behind this discordance. First, the pathogenicity assessment of the sequence variants was more challenging at the beginning of this century, given the lack of large population reference sequences. The frequency of predicted-to-be pathogenic variants carried by each individual, therefore, was yet unknown [43]. This led to the classification of many benign variants as pathogenic. Furthermore, the extreme genetic heterogeneity of rare autosomal recessive disorders (BBS, nephronophthisis) [44, 45] was unexpected. And the higher the number of the causal genes of a disease with a given prevalence, the higher the rate of heterozygous individuals in the general population. Thus, despite the rarity of these disorders, the rate of heterozygous individuals with a truly causal pathogenic variant is in the order of percentage. It is thus perfectly possible to identify accidentally heterozygous well-known pathogenic variants in a second locus without a causal role, not even speaking about the more frequent, predicted-to-be-pathogenic, but in reality benign variants. Whenever predicted or known pathogenic variants were identified in multiple genes, it was tempting to speculate that they act in concert, and the disorder has an oligogenic nature. However, when the mutational load was properly compared between the patient and the general population, it turned out that the second-locus variants are not more frequent in BBS or Joubert syndrome, indicating that oligogenism is at least not a common mechanism in these disorders [46, 47].

An example of a truly polygenic/multifactorial kidney disorder was nevertheless provided by *APOL1*-associated FSGS, a disease typical in populations with West African ancestry. The *APOL1* [S342G;I384M] (G1) and N388_Y389del (G2) alleles have evolved as the result of an evolutionary struggle against sleeping sickness caused by the parasite *Trypanosoma brucei rhodesiense*. Heterozygous individuals can neutralize this parasite, but at the cost of a lifetime FSGS risk of 4% in people with *biallelic* G1/G2 alleles [48, 49]. The risk is thus high, but substantially lower than the 100% seen in a Mendelian disease. In the case of an untreated HIV infection, the risk of HIV-associated nephropathy increases to 50% [49], demonstrating the decisive role of an environmental factor, unlike in a Mendelian disease.

The characteristics of Mendelian, oligogenic, and multifactorial disorders are summarized in Table 1.

**Table 1:** Characteristics of Mendelian and non-Mendelian disorders

Type of disorder	Mendelian	Oligogenic	Rare multifactorial
Familial clustering	Frequent*	Rare	Rare
Transmission pattern	Mendelian	Non-Mendelian	Non-Mendelian
Penetrance	Complete	Incomplete	Low
Prenatal/presymptomatic diagnosis	Possible	Risk can be estimated	Not possible
Relevance of the genetic tests in clinical practice	Decisive	High	Low
General population allele frequency of the variants	Rare	Rare	Both frequent and rare

*Unless AD disorder with early lethality or low reproductive fitness

## Mendelian vs. non-Mendelian in clinical practice

There is an inverse relationship between the number of the causal alleles and the risk of disease transmission. Since Mendelian disorders are determined by only one or two alleles, at-risk relatives are frequently affected. The significant familial clustering, giving the typical dominant and recessive transmission patterns, made the monogenic origin of specific disorders recognizable long before the identification of the causal genes.

In contrast, uncommon multifactorial disorders, determined by numerous alleles (and also by environmental factors), rarely cluster in families, as the risk of disease transmission is significantly lower. The different familial clustering of monogenic and rare multifactorial disorders can help their differentiation in clinical practice. Distinguishing the monogenic Alport from the multifactorial IgA nephropathy or the monogenic podocytopathy from the immune-mediated nephrotic syndrome [50] can be markedly improved by knowing the family history or potentially by investigating the first-degree relatives. Their affected status is strongly supportive of a monogenic origin.

Importantly, more frequent polygenic disorders, like CAKUT with an incidence of 1:300–2400 [51–53], may affect several members of a family even without a monogenic origin. Influenced by genetic, epigenetic, and environmental factors [54], its higher incidence together with a monogenic origin in at least 15% of the families [54] explains the relatively high proportion of affected first-degree relatives (4–25%) of patients with CAKUT [55, 56].

## Inbreeding and risk of homozygosity

Mendel performed cross-pollination only when crossing two pure lines. Their offspring, the hybrids, and the subsequent generations self-pollinated themselves, increasing the degree of inbreeding with each generation. Thus, the proportion of the “hybrids” decreased with each generation and the recessive phenotype became practically as frequent as the dominant after a few generations [6].

Inbreeding (self-pollination) does not change the allele frequency of the dominant and recessive alleles (assuming no natural selection), but does increase the relative frequency of the recessive phenotype from ¼ (*q*^2^) in the offspring of the hybrids (no inbreeding) to ~ ½ (*F* × *q*) in complete inbreeding, where *q* (ratio of the recessive allele) = ½ (since the two alleles were equally frequent in Mendel’s experiments), and *F* is the degree of inbreeding. After several self-pollinating generations (complete inbreeding), the value of *F* approaches 1.

In Mendel’s experiments, the proportion of the recessive phenotype only doubled secondary to inbreeding, since *q* was high (½), and therefore, the value of *q*^2^ and that of (*F* × *q*) did not differ by orders of magnitude. Similarly, in frequent autosomal recessive disorders, like in ARPKD, where *q* ~ 1/70 [57], the risk to the offspring of first-degree cousins (*F* = 1/16) to be affected secondary to inbreeding (*F* × *q* = 1/16 × 1/70) is only four times higher than the risk in a non-inbred population (*q*^2^ = 1/70^2^). Accordingly, most of the children with ARPKD are compound heterozygous (and not homozygous) for the causal variants in populations with a low rate of consanguinity. In contrast, in extremely rare autosomal recessive disorders, where *q* < 0.001, parental consanguinity increases the risk of the child by orders of magnitude (*F* × *q* ≫ *q*^2^). This explains why most patients with the extremely rare *PLCE1*-associated nephrotic syndrome are homozygous for the causal variant [28–30]. Thus, the less frequent an autosomal recessive disease is, the higher is the increase in the risk caused by parental consanguinity and the more important it is to explore the potential presence of inbreeding. Therefore, whenever a child is consulted with an unknown (= extremely rare) and potentially autosomal recessive disorder, one of the first questions should aim at parental consanguinity.

## Mendelian or non-Mendelian at the variant level

Transmission pattern is generally considered to depend on the causal gene and the phenotype. However, as several examples show above, pathogenic variants of the same gene may lead to different transmission patterns and may even change whether the disease is Mendelian (= completely penetrant) or non-Mendelian (= incompletely penetrant) (Table 2). It is also important to notice that different phenotypes related to the same variant may have different penetrances; i.e., while the nephronophthisis caused by homozygous *NPHP1* deletion is considered to be completely penetrant, retinitis pigmentosa or Joubert syndrome affects only a minority of the patients [40, 58, 59]. In summary, the proper description of the transmission pattern requires the knowledge of the gene, the variant, and the phenotype (Table 2).

**Table 2:** Transmission and penetrance in function of the causal variant and the phenotype

Gene, variant	Phenotype	Inheritance	Penetrance
*NPHP1*, homozygous deletion	nephronophthisis	Mendelian, AR	100%
	Joubert syndrome	oligogenic/multifactorial	~ 10%
*NPHS2* [p.R138Q];[p.R138Q]	SRNS	Mendelian, AR	100%
*NPHS2* [p.R229Q];[p.A284V]	FSGS	Mendelian, mutation-dependent AR	100%
*NPHS2*, heterozygous p.L330Vfs*15	FSGS	Mendelian, AD	100%
*PLCE1*, homozygous p.R2150*	SRNS	incompletely penetrant, AR	70–100%
*DKC1*, hemizygous p.E206K	SRNS	Mendelian, XR	100%
*DKC1*, heterozygous p.E206K	cataracts	incompletely penetrant, XD	< 100%
*APOL1*, biallelic G1 or G2 alleles	FSGS	multifactorial	4%
*CLCNKB*, homozygous deletion	Bartter syndrome	Mendelian, AR	100%
biallelic LOF variants in both *CLCNKA* and* CLCNKB*	deafness	digenic	100%
*COL4A5*, heterozygous LOF variant	Alport, CKD stage 5 in females	incompletely penetrant, X	< 40%
*COL4A5*, hemizygous LOF variant	Alport, CKD stage 5 in males	Mendelian, X	100%
*COL4A5*, hemizygous Gly624Asp	Alport, CKD stage 5 in males	incompletely penetrant, X	≪ 100%
*PKD1* LOF variant	ADPKD	Mendelian, AD	100%
*PKD1* R3277C	PKD	Mendelian, AR	100%
*PKHD1* T36M	ARPKD	Mendelian, AR	100%
*PKHD1* I2331K	ARPKD	incompletely penetrant, AR	< 100%

*AD* autosomal dominant, *AR* autosomal recessive, *ADPKD* autosomal dominant polycystic kidney disease, *ARPKD* autosomal recessive polycystic kidney disease, *CKD* chronic kidney disease, *FSGS* focal segmental glomerulosclerosis, *LOF* loss-of-function, *PKD* polycystic kidney disease, *SRNS* steroid-resistant nephrotic syndrome, *X* X-linked, *XD* X-linked dominant, *XR* X-linked recessive [14, 15, 20, 24, 26, 28, 31, 40, 48, 49, 59–66]

## Distinction of Mendelian and non-Mendelian variants

For genetic counseling and risk calculation of a Mendelian disorder, it is essential to distinguish the incompletely penetrant (non-Mendelian) variants and to determine their penetrance. This is easier to implement in dominant disorders where segregation analysis of affected families can help in the rough estimation of the penetrance, based on the proportion of unaffected family members with the causal variant.

Penetrance estimation is more challenging in autosomal recessive disorders for several reasons. Segregation analysis is less indicative of incomplete penetrance given the low number of at-risk family members. The underlying pathophysiology, a complete or almost complete loss-of-function makes the chance for an incomplete penetrance lower. Accordingly, incomplete penetrance with biallelic loss-of-function variants is exceptional: the early-onset *PLCE1*-associated nephrotic syndrome was only accepted after the identification of several families with non-penetrant phenotypes [28–30]. This suggested a non-Mendelian, oligogenic origin.

In the rare occasions where an autosomal recessive disorder is incompletely penetrant, it is generally associated with hypomorphic variants [60, 67, 68]. The identification of the incompletely penetrant variants can be greatly aided by knowing their allele frequencies in the general population. The term “maximum credible allele frequency” was proposed based on the general population allele frequency of the major pathogenic variant in a specific gene [69]. Pathogenic variants that are more frequent in the general population than the major variant of the patient cohort are expected to be benign or non-Mendelian. Nevertheless, this approach has limitations, both because rare variants may also be incompletely penetrant, and frequent variants can also be completely penetrant when being subject to interallelic interactions as in case of *NPHS2* R229Q, the allele frequency of which exceeds by more than an order of magnitude that of the major *NPHS2* R138Q variant in the general population.

We recently introduced a population-genetic algorithm to identify incompletely penetrant variants in autosomal recessive disorders and to calculate their penetrance based on the relative allele frequency of specific pathogenic variants to the loss-of-function variants in the patient and the general population [60]. The sensitivity of this approach strongly depends on the patient cohort size, and will be especially informative in large national patient cohorts. We identified novel incompletely penetrant variants in several autosomal recessive disorders, also the *PKHD1* S1833L or I2331K variants in ARPKD, supporting the notion that a non-negligible proportion of frequent variants in autosomal recessive disorders are in fact not Mendelian [60]. Knowledge and further investigation of these variants are essential for proper genetic counseling.

Nevertheless, whenever the degree of penetrance is estimated based on general population data, it is important to keep in mind that it can be dramatically different in families with affected children. Accordingly, the penetrance of severe liver fibrosis in patients with alpha1-antitrypsin deficiency secondary to *SERPINA1* homozygous p.E366K (often referred to as “Z” allele) was found in 3/122 (2.5%) in a prospective study screening 200,000 newborns [70], but an order of magnitude higher (8/20, 40%) in siblings of a severely affected child [71], reflecting the enrichment of additional risk factors in families with affected children. Thus, different penetrance data apply to families with and without affected children.

The less frequent a variant, the more challenging to estimate its penetrance, but it is expected to be greatly helped by the rapidly growing patient cohorts and exome and genome sequence data in the general population.

## The recessive researcher

Mendel had a shy, introverted character. He had a reputation for self-deprecating jokes. He published only two major scientific papers in his life, he had no pupils, and his work did not give rise to a novel school of science. No one during his lifetime cited his research, and his work was only discovered several years after his death. He never qualified as a teacher and was thus categorized as a substitute teacher for all his career [2]. Darwin, the other great biologist of the nineteenth century, was famous already in his own lifetime. While he was in the forefront of nineteenth-century science, Mendel was hiding in the background with no patent, no citation, no reputation, and no “research grant.” And even if scientific grants had existed and he had had the opportunity to apply for one, who would have financed the research of a scientist with two publications and zero citations? Mendel was thus only recompensed by his own intellectual excitement, a currently slowly fading source of pleasure. However, pure intellectual excitement was enough reward for Mendel to complete a highly meticulous work that laid the foundations of genetics.

After his election as abbot of the Augustinian monastery, he had less time to continue his research [2]. The pea weevil regained his territories in the evolutionary struggle, and thus, the Mendelian ratios of the characters must have been lost. The work was however done, even if its importance was discovered more than a decade after Mendel’s death [2].

Mendel suffered from “chronic nephritis,” and according to his recently obtained genome sequence data, he carried some risk variants for “kidney diseases” [72]. These variants must be unique in the field of nephrology, as they are both Mendelian and non-Mendelian.

The recessive (reclusive) Mendel transmitted his dominant findings to the next generations on a few pieces of paper allowing them to understand the basics of genetics. Genetic research has undergone a major burst afterwards and 100 years after Mendel’s landmark publication (1866), the genetic code was already cracked [73].

## Key summary points

1. The constant 3:1 ratio among the offspring of the hybrids allowed for Mendel to understand that binomial traits are determined by distinct elements.
2. The dominant or the recessive terms, as introduced by Mendel, in general apply to all disease alleles of a specific gene. Several exceptions have been however identified. Therefore, the transmission pattern should be described in function of the causal variants and the specific organ involvement.
3. Familial clustering can help the differentiation of Mendelian and rare multifactorial disorders in clinical practice.
4. The less frequent an autosomal recessive disease is, the more important is the effect of inbreeding on its occurrence.

## Multiple choice questions with answers

1. What are the major consequences of Mendel’s work in the clinical practice? (select all that apply)
  - The monogenic origin of a disorder can be established based on the transmission pattern.
  - The number of the phenotype determining ‘elements’ (alleles) in an individual could be determined.
  - The role of inbreeding in the rate of the recessive phenotype could be understood.
  - Examples of digenic inheritance have been provided.
2. A boy is diagnosed with nephrotic syndrome at the age of 8 months. His twin sister is also found to have nephrotic range proteinuria. The healthy parents are first-degree cousins. What would you do?
  - As the siblings are twins, their affected status should not be considered familial clustering. Steroid therapy should be started, and genetic test to be done only in case of steroid-resistance.
  - Based on the familiar clustering in a consanguineous family, the monogenic origin is evident. Steroid therapy is useless, as well as the genetic test.
  - Start a genetic test and steroid treatment of the index child.
  - A genetic test is necessary, but as the monogenic origin is highly suspicious, no steroid therapy to be initiated.
3. An 8-year-old girl is diagnosed with isolated nephronophthisis secondary to homozygous *NPHP1* deletion. What is the risk of her 1-month-old asymptomatic brother to develop nephronophthisis and what is his risk of Joubert syndrome?
  - 50% and negligible.
  - 25% and < 3%
  - 25% and < 10%
  - 10% and 5%
4. A 30-year-old woman was clinically diagnosed with IgA nephropathy based on subnephrotic proteinuria (0.5–1 g/d) and microscopic hematuria. Her two-year-old son was recently diagnosed with similar degree of proteinuria and hematuria and the twin sister with isolated hematuria. No complaint of hearing impairment in any of them. What would you do? (select all that apply)
  - Reassessment of the mother’s diagnosis. Monogenic origin is suggested.
  - Genetic test for Alport syndrome.
  - Kidney biopsy in both children.
  - Start immunosuppression in the son.
